# Supplementary material for: Transcriptional Profiling of the Candida auris Response to Exogenous Farnesol Exposure
Source: mSphere. 2021 Oct 13;6(5):e00710-21. doi: 10.1128/mSphere.00710-21 (PMC8513684; doi:10.1128/mSphere.00710-21)
Supplement: TABLE S1 [file msphere.00710-21-st001.docx]

**Supplementary Table 1:** Oligonucleotide primers used for RT-qPCR analysis

B9J08_000486 (*ACT1*)

5´-CTTGTTCCCAGGTATTGC-3´and 5´-CTCGTCGTATTCTTGCTTG-3´

B9J08_004331 (*ADH1*)

5´-CTCGCTGCTCCCATCTTG-3´and 5´-TCGTCACCACCGTCAATG-3´

B9J08_002298 (*CAT1*)

5´-GTGCCTACGGTGTCTTTG-3´and 5´-TTGGATGGGTCTCTGATG-3´

B9J08_000164 (*CDR1*)

5´-TTGGAGATGGGTGCTTAC-3´and 5´-GCTGAAGGTTGATGGATG-3´

B9J08_003981 (*MDR1*)

5´-CGACCAAGACCAACCACATTC-3´and 5´-CGTATCCGAACACAAAAAGCG-3´

B9J08_004309 (*PFK1*)

5´-CGCCTACTCTTCTTTGGAC-3´ and 5´-ACGGTGGTGTTTCTCTTTC-3´

B9J08_001458 (*RBT1*)

5´-AGGTAACGGTGGCAAAGGTG-3´and 5´-AAGGCAGCAGCAAGCAAAG-3´

B9J08_002251 (*HGT2*)

5´-TACCTCCCCAGAACCATC-3´ and 5´-AATAGTGCCCCAAAAGTCC-3´

B9J08_001484 (*POT1*)

5´-CCCTTCCTCCAAACAGTC-3´ and 5´-AATACCAGCAGCCAAACAG-3´

B9J08_005429 (*NRG1*)

5´-GTGCCTTATCAGAACATTGC-3´and 5´-GCCCAGTGTAGTGAACAGAC-3´

B9J08_002231 (*PCD11*)

5´-TGTTTGTCGGTGATGGTTC-3´ and 5´-TGGCTGGATGTCGTTGTAG-3´

B9J08_002108 (*FTR1*)

5´-ACGGTTCTGTCCTTTCCTAC-3´ and 5´-CTTCTTGCCCTTGATATGG-3´

B9J08_002762 (*INO1*)

5´-TTCCTTGTTGACGCTGGTATC-3´and 5´-CGACATCCTTTCCCTTCTTG-3´

B9J08_000261 (*ERG1)*

5´-GCATTGTACTCGTTATTCGC-3´ and 5´-ACCCTTCTCGCAGTAGTTG-3´

B9J08_002366 (*CCP1*)

5´-ACTGGGTGCCTATGTTCGTAAG-3´and 5´-GTTTCATCATCCTGGTATTGCC-3´
